# Identification and Characterization of a Dual-Acting Antinematodal Agent against the Pinewood Nematode, *Bursaphelenchus xylophilus*

**DOI:** 10.1371/journal.pone.0007593

**Published:** 2009-11-11

**Authors:** Wan-Suk Oh, Pan-Young Jeong, Hyoe-Jin Joo, Jeong-Eui Lee, Yil-Seong Moon, Hyang-Mi Cheon, Jung-Ho Kim, Yong-Uk Lee, Yhong-Hee Shim, Young-Ki Paik

**Affiliations:** 1 Yonsei Proteome Research Center and Department of Biochemistry, College of Life Sciences and Biotechnology, WCU Program, Yonsei University, Seoul, Korea; 2 South Forest Research Center, Jinju, Gyeongsangnam-do, Korea; 3 Hanwha Chemical Research and Development Center, Taejon, Korea; 4 Department of Bioscience and Biotechnology, and Bio/Molecular Informatics Center, Konkuk University, Seoul, Korea; Research Institute for Children and the Louisiana State University Health Sciences Center, United States of America

## Abstract

The pinewood nematode (PWN), *Bursaphelenchus xylophilus*, is a mycophagous and phytophagous pathogen responsible for the current widespread epidemic of the pine wilt disease, which has become a major threat to pine forests throughout the world. Despite the availability of several preventive trunk-injection agents, no therapeutic trunk-injection agent for eradication of PWN currently exists. In the characterization of basic physiological properties of *B. xylophilus* YB-1 isolates, we established a high-throughput screening (HTS) method that identifies potential hits within approximately 7 h. Using this HTS method, we screened 206 compounds with known activities, mostly antifungal, for antinematodal activities and identified HWY-4213 (1-n-undecyl-2-[2-fluorphenyl] methyl-3,4-dihydro-6,7-dimethoxy-isoquinolinium chloride), a highly water-soluble protoberberine derivative, as a potent nematicidal and antifungal agent. When tested on 4 year-old pinewood seedlings that were infected with YB-1 isolates, HWY-4213 exhibited a potent therapeutic nematicidal activity. Further tests of screening 39 *Caenorhabditis elegans* mutants deficient in channel proteins and *B. xylophilus* sensitivity to Ca^2+^ channel blockers suggested that HWY-4213 targets the calcium channel proteins. Our study marks a technical breakthrough by developing a novel HTS method that leads to the discovery HWY-4213 as a dual-acting antinematodal and antifungal compound.

## Introduction

Pine wilt disease, caused by the mycophagous and phytophagous pinewood nematode (PWN), *Bursaphelenchus xylophilus*, is a great threat to pine forests, imposing a socioeconomic burden worldwide [1]–[4]. The most serious damage has been incurred in northeastern Asian countries, including Japan, China, Taiwan and Korea, where indigenous pine trees, such as *Pinus densiflora, Pinus thunbergii*, and *Pinus massoniana*, are highly susceptible to PWNs. PWN infection and associated damage have also expanded to other conifers and oak trees in Korea [5]. It is well established that PWNs feed on the hypha of the dimorphic fungi, such as *Botrytis cinerea, Ophiostoma minus*, and *Ceratocystis spp*, and are introduced into the shoots of trees by the beetle, *Monochamus spp.*, by adult insect vectors feeding on twigs of healthy pine trees (maturation feeding), or by adult females ovipositing on freshly cut timbers or dying trees [2], [6]–[8]. This suggests that an ideal antinematodal agent would possess both antifungal and nematicidal functions for effective control of PWN. However, there has yet to be such a breakthrough and no such dual-function therapeutic antinematodal agent has been developed to control PWN and the pine wilt diseasse. Although several chemical agents are presently available to prevent PWN infection, including morantel tartrate, avermectin and emamectin benzoate [9]–[11], each is of limited value due to poor water solubility, lack of therapeutic efficacy and/or high cost. Thus, there is an urgent need for the development of new therapeutic antinematodal agents that circumvent these problems.

Ideally, a new trunk-injection antinematodal drug would be highly soluble in water and possess dual nematicidal and antifungal activity, thus killing not only the nematode but also the xylem-dwelling dimorphic fungi that serve as its food source [2], [6]–[8], [12]. Here, we report the development of a new HTS method and its use to demonstrate proof-of-principle of the novel concept of multi-targeted antinematodal agents. This work has led to the identification of potential antinematodal candidate HWY-4213, a highly water soluble protoberberine derivative that is effective against *B. xylophilus*, whose discovery serves as a valuable starting point for the development of a commercial agent with the promise of potentially eradicating the deadly pine wilt disease.

## Results

### Life Cycle of *B. xylophilus* YB-1 Isolates

**Figure 1:**
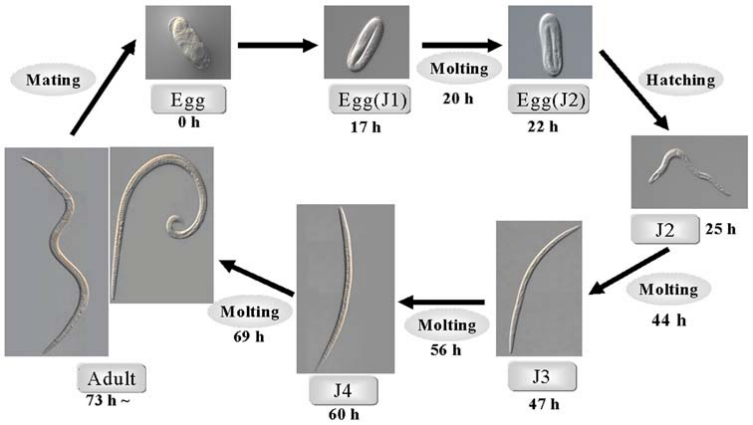
Life cycle of *B. xylophilus*. *B. xylophilus* life cycle was examined in worms grown on plates of *Botrytis cinerea* as described under ‘Materials and Methods’. A hundred adult female *B. xylophilus* were placed on an observation plate and allowed to lay eggs for 3 h at 25°C, before being removed. Details on the morphological changes are shown in Fig. S1.

To investigate the basic physiological characteristics of YB-1 isolates, we examined its life cycle by measuring the duration of each developmental stage. As summarized in Fig. 1 and Table 1, when grown in fungal cultures of *B. cinerea* at 25°C, the life cycle of the YB-1 isolates was about three days, similar to that of the virulent S-10 and T4 isolates but shorter than the avirulent isolates (e.g., OKD-1, C14-5) [13]. The pattern of early embryogenesis of the *B. xylophilus* YB-1 isolate, originally obtained from Busan, Korea, was similar to that of the S-10 isolate originally obtained from Shimane Prefecture, Japan [14]. They molted from J1 to J2 inside eggs and hatched at the J2 stage, and the well-developed J4 soon molted to adults. Adult females were slightly longer than males but trunk thickness was the same in both genders (0.25 mm). Shortly after the final molt, adult females began to lay eggs. The average duration of egg laying and mean fecundity per adult YB-1–isolate female (mean±S.D.) were 14±2 (n = 60) and 149±25 (n = 60), respectively, which are similar to those of the S-10 and T4 isolates [13]. In general, virulent isolates have a longer egg-laying period and produce more eggs than avirulent isolates [13]. When grown at 25°C, *B. xylophilus* had a mean life span (MLS) of approximately 15 days and a maximum life span (XLS) of approximately 30 days (Table 2). The life-cycle data presented in Fig. 1 and Table 1 may be the first of its kind in which the details of all developmental stages were well observed in this virulent *B. xylophilus*.

**Table 1:** Life cycle and body size of *B. xylophilus* (YB-1 isolate).

											Adult	
	Egg	Egg (J1)	Molting	Egg (J2)	J2	J2 molting	J3	J3 molting	J4	J4 molting	F*	M†
Time (h)	0	17	20	22	25 (±1.0)	44 (±1.0)	47 (±1.0)	57 (±1.0)	60 (±1.0)	70 (±2.0)	74 (±2.0)	74 (±2.0)
length (µM)	54.7 (±0.4)	59.5 (±0.6)	59.6 (±0.5)	60.1 (±0.7)	186.4 (±19.4)	248.4 (±5.1)	405.6 (±15.2)	450.2 (±7.2)	610.4 (±50.1)	711.7 (±35.1)	1015.2 (±102.1)	890.4 (±98.2)
width (µM)	22.1 (±0.4)	24.3 (±0.3)	24.2 (±0.3)	24.3 (±0.4)	10.4 (±0.4)	12.1 (±0.5)	13.1 (±0.5)	15.3 (±0.6)	16.3 (±0.5)	19.8 (±0.7)	27.3 (±7.2)	21.8 (±5.1)

*Female.

† Male. Values are mean±SD (n = 20) at 25°C, from three independent experiments.

**Table 2:** Basic characteristics of *B. xylophilus* (YB-1 isolate).

			Mean Life span			
			mated		non-mated	
Physiological properties of YB-1 isolates*	**Average duration of egg laying (days±SD, n = 60)**	**Mean fecundity per adult female (mean±SD, n = 60)**	**Male**	**Female**	**Male**	**Female**
Basic Characteristics	14±2 days	149±25 eggs	17 days	18 days	45 days	51 days

*Worms obtained at a certain stage were grown to young adults. One female and three males were placed on each observation plate and cultured at 25°C for several days.

### Influence of Mating on the Life Span of *B. xylophilus*

From the point of view of pest management, it would be useful to determine the life spans of both mated and unmated groups. Since *B. xylophilus* is a dioecious organism, mating may be an important part of its life cycle with respect to reproduction. Reasoning that mating might have an effect on longevity, we measured the life span of mated and non-mated groups respectively. In the continuously mated group, the MLS of *B. xylophilus* at 25°C was 17 days for males and 18 days for females, and the XLS for both sexes was 30–35 days (Table 2, Fig. S2 *A*). The MLS of the non-mated male and female was 45 days and 51 days, respectively, and the XLS of both sexes was 70 days (Fig. S2 *B*). Thus, the MLS of the non-mated *B. xylophilus* is more than 2.5*-*fold longer than that of their mated counterpart and the XLS is approximately 2-fold longer. In general, females live longer than males in both mated and non-mated groups.

### Establishment of High-Throughput Screening (HTS) for Nematicidal Agents

**Figure 2:**
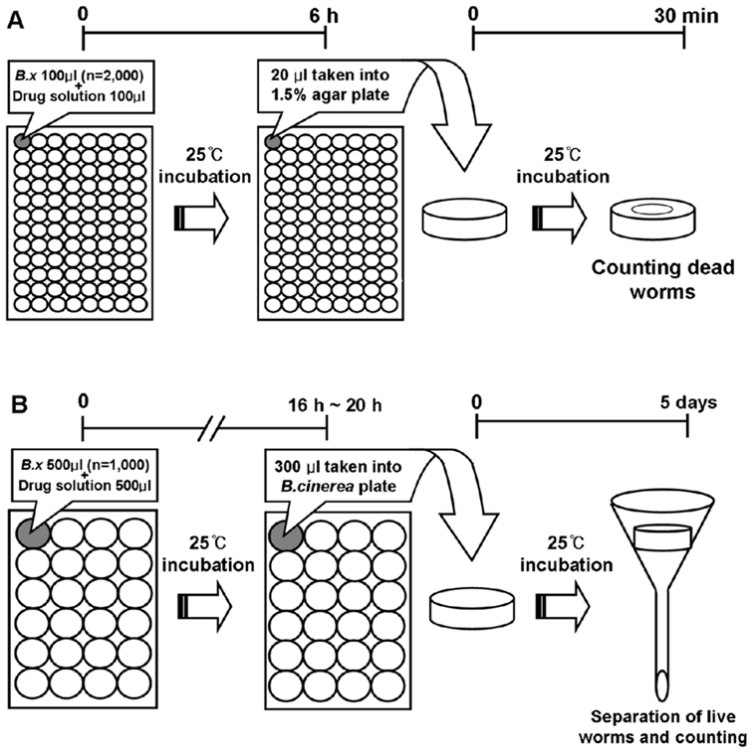
Summary of the high throughput screening method. (***A***). A stock solution of *B. xylophilus* was prepared with a concentration of approximately 10,000 nematodes/ml. An aliquot of *B. xylophilus* was dispensed into 96-well plates with a concentration of approximately 2000 nematodes/100 µl per each sample. An aliquot (100 µl) of diluted stock solution (200 mM) of the testing compounds was added onto each well and the treated nematodes were grown at 25°C for 6 h. After incubation, 20 µl of the sample was taken into 1.5% agar plate and the number of dead worms counted for measurement of LD50 while sitting at 25°C. If necessary, the nematicidal activity of those selected drugs (e.g., HWY-4213) was verified using cotton ball analysis (CBA). (***B***). The CBA was carried out as described [9]. Briefly, *B. xylophilus* are reacted with the drugs contained in cotton boll tips for 16 to 20 h as described [9] and then the drug-treated *B. xylophilus* on a plate were treated with a mold used as a feed are cultured at 25°C for 5 days. After 5-day incubation, the drug effects are assessed by measuring the reproductive rates.

There has been no standard screening system available for the identification of antinematodal agents for *B. xylophilus*. The cotton ball assay (CBA) has been the mainstay for screening preventative antinematodal agents [9], which was found to be impractical for screening the nematicidal agents against *B. xylophilus*. Therefore, we sought to design a HTS method that would allow several hundred compounds to be rapidly screened for either preventative or therapeutic usage (Fig. 2). In this method, about 1000 *B. xylophilus* are treated with either the testing compounds or control agents (e.g., morantel tartrate) utilizing the 96-well plates. We determined the relative efficacy of the agents by measuring LD50. We found that the HTS method provided initial results within 6–7 h, and it can directly measures the antinematodal effect of the testing agent (see below).

### Identification of HWY-4213 as a Potent Lead of Antinematodal Agent

In the initial screen, we used a chemical library containing 206 highly selective compounds (data not shown), most of which had been previously shown to exhibit the anti-fungal activity by inhibiting sterol or chitin biosynthesis [15], [16]. We also included several well-known commercial preventive antinematodal agents, such as morantel tartrate, in the assay as controls. Four compounds (HWY-4213, HWY-5038, HCI-15014, and HCI-15176) were initially identified and were selected for further analysis. Among the compounds isolated, HWY-4213 (1-*n*-undecyl-2-[2-fluorphenyl] methyl-3, 4-dihydro-6, 7-dimethoxy-isoquinolinium chloride) exhibited the most potent antinematodal activity (LD50 = 447 µM) which was more potent than control agents such as morantel tartrate (LD25 = >15.602 mM). Representative examples of the major screening results are summarized in Table 3. Moreover, HWY-4213 also showed a potent nematicidal activity against *C. elegans* (LD25 = 0.082±0.059 [SD] mM) and *C. briggsae* (LD25 = 0.076±0.042 [SD] mM) in three independent experiments [n = 3]) (Table S1). These results confirmed that HWY-4213 is a promising candidate nematicidal agent that warrants further testing for both the therapeutic and preventative applications. Since morantel tartrate showed slightly better solubility than other control agents such as levamisol hydrochloride, emamectin benzoate, and abamectin (data not shown), we decided to use it as a control in all subsequent experiments.

**Table 3:** Anti-nematodal effects of various compounds with known modes of action.

Test compounds†	Class	High-Throughput Screening* (mM)		
		LD25	LD50	LD95
HWY-4213	Fungicide	0.171 (±0.089)	0.447 (±0.079)	0.944 (±0.002)
HWY-5038	Fungicide	0.366 (±0.092)	0.748 (±0.085)	1.442 (±0.093)
HCI 15014	Fungicide	1.144 (±0.115)	>2	>2
HCI 15176	Fungicide	1.337 (±0.098)	>2	>2
Morantel tartrate‡	Muscle activity blocker	15.602 (±1.718)	>50	>50

*Values are mean±SD (n = 3).

† Each drug was tested over a 0–2 mM concentration range, except controls, which were tested at 0–4 mM (emamectin benzoate, abamectin and levamisol hydrochloride) or 0–50 mM (morantel tartrate,).

‡ Since other control chemicals (*e.g*., emamectin benzoate, abamectin, levamisol hydrochloride) showed extremely low solubility which prevents preparation of the stock solution in DMSO, we were not able to get their LD50 values even at greater than 4 mM of each agent used under this assay condition.

### Antifungal Activity of HWY-4213 against *Ophiostomatoid minus* Fungi

As part of our study for mode of action, we were interested in testing whether HWY-4213 possess a potential antifungal activity against the blue stain fungus *Ophiostomatoid minus*, one of several fungi that *B. xylophilus* feeds during the mycophagous phase [8], [12]. Ideally, an agent with the potent antinematodal activity against the causative agent of the pine wilt disease, would also have a antifungal activity to effectively control the xylem-residing nematodes. Accordingly, we performed antifungal susceptibility tests of HWY-4213 and a number of currently available preventive antinematodal agents by measuring the minimal inhibitory concentrations (MIC) and the minimal fungicidal concentrations (MFC). *Aspergillus flavus* and *A. fumigatus* were selected as fungal reference strains in these tests. As summarized in Table 4, the MIC and MFC values for HWY-4213 were 2.0 mg/L and 4.0 mg/L, respectively, which are similar to those for miconazole and ketoconazole [16]. In contrast, the currently available preventive antinematodal agents, such as emamectin benzoate, abamectin, ivermectin and morantel tartrate, showed no antifungal activity.

**Table 4:** Anti-fungal activity of the representative antinematodal agents*.

	*Ophiostom-atoid* fungi		*A. flavus*		*A. fumigatus*	
The representative antinematodal agents	MIC (mg/L)	MFC (mg/L)	MIC (mg/L)	MFC (mg/L)	MIC (mg/L)	MFC (mg/L)
HWY-4213	2.0	4.0	16.0	16.0	8.0	8.0
Amphotericin B	0.25	0.5	0.5	1	0.5	1
Itraconazole	0.125	0.25	0.25	0.5	0.25	1
Miconazole	1.0	4.0	8	8	8	8
Ketoconazole	2.0	4.0	>16	>16	16.0	>16
Fluconazole	>32	>32	>32	>32	>32	>32
Emamectin benzoate	>32	>32	>32	>32	>32	>32
Abamectin	>32	>32	>32	>32	>32	>32
Ivermectin	>32	>32	>32	>32	>32	>32
Morantel tartrate	>32	>32	>32	>32	>32	>32

*Only one representative data is shown here. This experiment was repeated four times.

### Preventative and Therapeutic Effects of HWY-4213 against Pot-Grown Pine Trees

**Figure 3:**
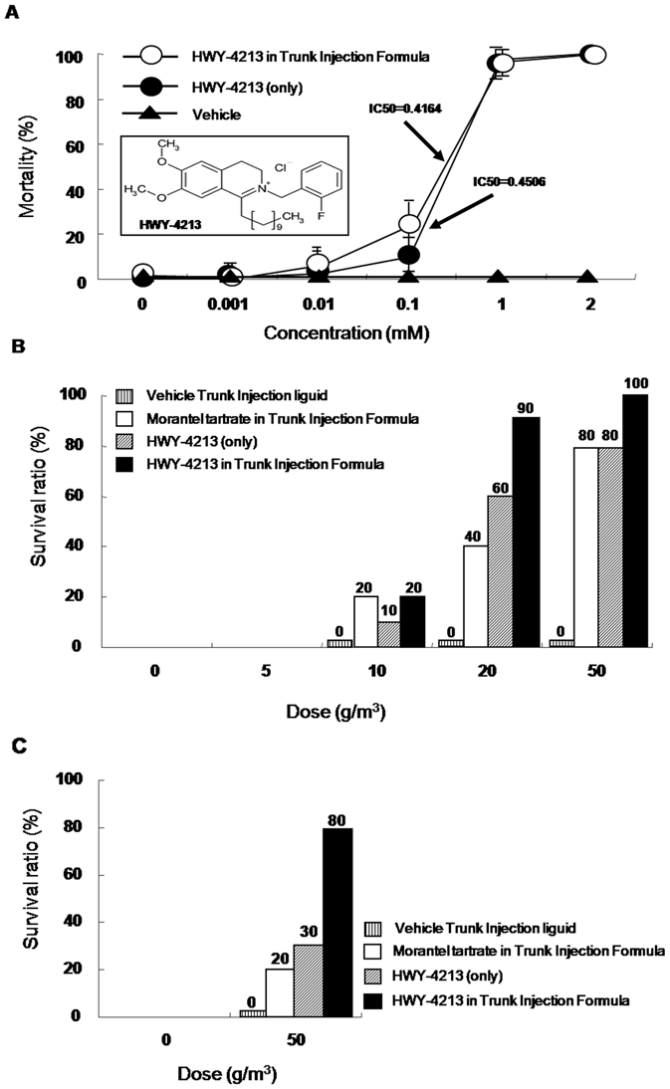
Evaluation of *in vivo* preventative and therapeutic effects of HWY-4213 against PWN in pot-grown pine trees. (*A*) The nematicidal effect of HWY-4213 formulated as a trunk-injection agent was evaluated using the *in vitro* HTS assay. (*B*) The *in vivo* preventative activity of HWY-4213 in trunk-injection formula was examined using 4 year old pot-grown pine trees. In this experiment, pine trees were incubated for 2 weeks after receiving different doses (10 pine trees/dose) of one of the testing compounds: HWY-4213 only, HWY-4213 in trunk-injection formula or morantel tartrate in trunk-injection formula. After this 2-week pretreatment, the trees were inoculated with *B. xylophilus* (10,000 nematodes/mL) and maintained in pots for 2 months, during which their growth status was observed. Trees that died were cut into pieces and observed for the presence of *B. xylophilus*. (*C*) The *in vivo* therapeutic activity of HWY-4213 in trunk-injection formula was measured at a single dose of 50 g/m^3^. Ten pine trees were first inoculated with *B. xylophilus* (10,000 nematodes/mL) and then incubated for 2 weeks. Thereafter, each PWN-infected tree received a single injection of each drug (50 g/m^3^) and was maintained in the pot for 2 months, during which the dead trees were observed. To confirm that dead trees were infected with PWN, they were cut into pieces and examined for the presence of *B. xylophilus*.

Before applying HWY-4213 as a treatment for PWN-infected trees, we needed to find an appropriate carrier solution with which HWY-4213 could be blended for tree injections. To rescue the pinewoods with pine wilt diseases, it is important that the injected anti-nematodal compound be rapidly and widely dispersed once applied. In addition, a principle trunk-injection agent should have good water solubility to facilitate effective diffusion of the antinematodal ingredients throughout the plants (Table S2). Therefore, selection of a carrier solvent for trunk-injection agent should be based on the solvent's freezing point, appearance and resin solubility. A preliminary screen showed that a solution containing 200 µl methyl ethyl ketone (MEK) had a higher resin solubility and a lower freezing point than other carriers (Table S2, see also Text S1 for details on the methods) and relatively short injection time (Table S3). Thus, MEK was selected as the carrier solvent for HWY-4213 in *in vivo* tests. To test the nematicidal (in MEK) effectiveness of HWY-4213, an *in vitro* HTS assay was carried out. As anticipated, the liquid formula of HWY-4213 containing MEK (20% v/v) (IC50 = 0.4164 mM) had essentially the same *in vitro* nematicidal dose-response curve as HWY-4213 alone (Fig. 3A; IC50 = 0.4506 mM), suggesting that the presence of MEK did not diminish the nematicidal activity. To investigate the *in vivo* activity of HWY-4213, we used pot-grown 4-year-old pinewood seedlings. In the control group, we observed the initial symptoms of wilting disease, such as leaf yellowing, 2 weeks after inoculation of *B. xylophilus* (10,000 nematodes/mL) (Fig. S3). As shown in Figure 3B, the MEK-containing liquid formula of HWY-4213 administered as a trunk injection was much more effective than morantel tartrate in preventing the wilting of pre-infected (10,000 nematodes/mL), pot-grown 4-year-old pine trees. This effect appears to exhibit in a dose-dependent manner up to 50 g/m^3^ [10]. When tested *in vivo* at a fixed dose of 50 g/m^3^, this liquid HWY-4213 formulation rescued 80% of tree wilting; HWY-4213 alone and morantel tartrate, tested at the same dose, and reduced wilting by approximately 30% and 20%, respectively (Fig. 3C). Both the preventative and therapeutic potential shown by the HWY-4213 liquid formula deserves further exploration for development of a broad-spectrum anti-nematodal agent.

### Potential Mechanism of Action for HWY-4213

**Figure 4:**
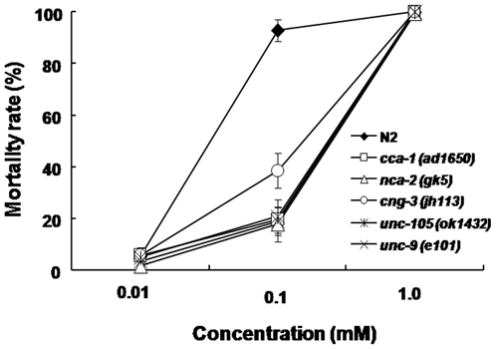
Screening of *C. elegans* mutants in search of nematicidal targets of HWY-4213. Total 39 *C. elegans* mutants that are known to be deficient in channel proteins were tested for their resistance to HWY-4213 using the HTS method. Five mutant strains that are associated with either calcium channel or muscle genes are shown to be resistant to HWY-4213.

**Figure 5:**
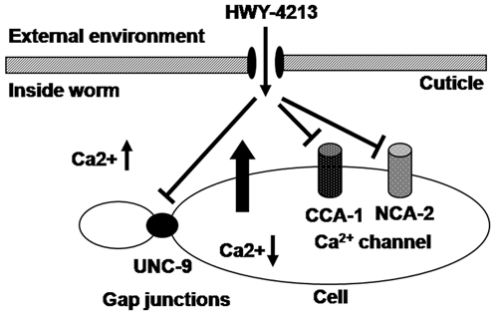
Working model for the nematicidal action mechanism by HWY-4213. Based on the primary screening results, we hypothesized that HWY-4213 kills the nematode *B. xylophilus* by inhibiting functions of certain calcium channel proteins as well as the UNC-9 protein in gap junctions, which could result in bursting of calcium ions. The exact mechanism of action involving this calcium channel blocking by HWY-4213 remains to be established.

We provided evidence that HWY-4213 acts as an antinematodal agent by killing both the nematode and feeding fungi in the mycophagous phase. Although the biochemical mechanism of antifungal action by HWY-4213 is predicted to be its specific inhibitory activity against sterol 24-metlytransferase [16, Paik et al., 2003, unpublished data], its nematicidal action mechanism is not known. Since HWY-4213 tends to kill nematodes within a short period of time (∼6 h) under our HTS condition, we thought that it might act on certain ion channel proteins such as Ca^2+^ channels. In fact, previously published studies have already shown that one of the pharmacological action of the berberine derivatives is its strong blocking action against mammalian Ca^2+^ channels [17]–[19]. Based on this information, we surveyed Ca^2+^ channel mutants for responses to HWY-4213. Given no mutants available for *B. xylophilus*, we selected 39 known channel gene mutants from *C. elegans* strains through *C. elegans* Genetics Center (CGC, Minneapolis, MN) (Table S4) and tested for their relative sensitivity to this agent. As shown in Fig. 4, we identified 3 genes involved in calcium channels (*cca-1*, *nca-2* and *cng-3*) and 2 genes in muscle controls (*unc-105* and *unc-9*) that are likely to be the targets of HWY-4213. To further identify which calcium channels are involved, we selected 10 different calcium blockers (nine were L-type, one was T-type) and tested their relative nematicidal activity against *B. xylophilus*. As summarized in Table 5, mibefradil (T-type) showed the highest lethal activity (36.8±5.7% lethality at 1 mM) followed by verapamil (L-type, 24.3±5.1% lethality). Interestingly, the morphological view of dead animals by 0.1 mM HWY-4213 was very similar to that by mibefradil but clearly different from that by verpamil (Fig. S3). These results suggests that HWY-4213 may have the nematicidal activity by blocking the calcium channel activity (more likely the T-type) of *B. xylophilus* (Fig. 5).

**Table 5:** Relative sensitivity of *B. xylophilus* to Ca^2+^ channel blockers.

Test compounds	Ca^+^ channel type	Lethality against *B. xylophilus* (%)*		
		0.01 mM	0.1 mM	1 mM
HWY-4213	-	0.0	21.1 (±2.5)	100.0
Amlodipine	L	0.0	0.0	0.0
Bepridil	L	0.0	0.0	9.8 (±2.4)
Diltiazem	L	0.0	0.0	2.5 (±1.2)
Felodipine	L	0.0	0.0	10.1 (±3.2)
Isradipine	L	0.0	0.0	5.6 (±2.6)
Nicardipine	L	0.0	0.0	0.0
Nifedipine	L	0.0	0.0	0.0
Nimodipine	L	0.0	0.0	0.0
Verapamil	L	0.0	0.0	24.3 (±5.1)
Mibefradil	T	0.0	0.0	36.8 (±5.7)

*Values are mean±SD (n = 3).

## Discussion

### Impact of a New HTS Development

The HTS method we described here proves to be faster and efficient in screening for potent nematicidal agents compared to the conventional CBA method when a large number of chemicals or efficacy assessments are required. For example, we would need one 24-well plate and a set of twenty-one 100-mm plates containing *B. cinerea*, all of which had to be continuously incubated for 6 days, if we were to use the CBA method. At this rate, screening of 200 plus samples would have taken almost one year. In addition to providing rapid results, the HTS method is also cost-effective and the results are highly reproducible. Because of the principle differences between CBA (to determine IC50 for growth inhibition) and HTS methodologies (to determine LD50 for nematicidal activity), a direct comparison of the two methods in compound efficacies does not appear to be practical, nevertheless.

### Chemical Properties and Biological Function of HWY-4213

HWY-4213, a semi-synthetic protoberberine (Fig. 1A) originally prepared as one of the anti-fungal agents inhibiting the sterol 24-methyl transferase [15], [16] of *Candida albicans*, was identified by our HTS method as a potent nematicidal agent. Its nematicidal and antifungal activities mark it a potentially strong candidate for development into a therapeutic and preventive nematicidal trunk-injection agent. In our preliminary study, we found that two positions (Z1 and Z5) of the benzene ring (one of the two functional groups) which bind to F and Cl appear to have a key role in its anti-nematodal effect. However, further studies are necessary. Thus, HWY-4213 not only is nematicidal, it also eliminates nematode's food source by exhibiting a potent antifungal agent against *O. minus*. Interestingly, the antifungal phytoalexin was detected in the PWN-resistant trees [20], suggesting that the elaboration of antifungal activity by trees may be a key to the resistance by the pine trees.

The physical properties of HWY-4213 represent another potential advantage. It has previously been reported that only those compounds with water solubility greater than 1000 mg/L prevented wilting in pine trees that had been artificially inoculated with PWN [10]. While currently available anti-nematodal compounds have very a low water solubility (e.g., 24 mg/L for emamectin benzoate) [9], the water solubility of HWY-4213 is greater than 100,000 mg/L. The high solubility of HWY-4213 allows it to be applied year-round regardless of the presence of pine oleoresin. Blending HWY-4213 with MEK created a liquid formulation that showed a even greater absorption rate than that of other formulation, while maintaining the full biological activity.

Finally, although HWY-4213 is a lead compound, not the final product for commercial usage, it would be nice to known its general toxicity. We have shown that [HWY-4213 exhibited no acute toxicity at >2,000 mg/kg body weight (oral) in Sprague Dawley Rats and negative for AMES test using *Salmonella typhimurium* [21].

### Preliminary Assessment for Mode of Action

We hypothesized that HWY-4213 may function by blocking the calcium channels based on the following observations. First, literatures showed supporting evidence that the berberine derivatives contain the strong blocking action against mammalian Ca^2+^ channels [17]–[19]. Second, we observed that HWY-4213 resulted cell lysis was similar to that by miberfradil (T-type Ca^2+^ channel blocker, 1 mM) (Fig. S4). Moreover, we found that the nematodes with mutations in the genes encoding the calcium channel alpha 1 subunit, *cca-1(ad1650)* [22], [23] and four-domain alpha 1U Ca^2+^ channel subunit, *nca-2(gk5)*, were more likely to be resistant to HWY-4213. Apparently, the T-type Ca^2+^ channel blockers are generally known to have higher lethality than the L-type Ca^2+^ channel blocker (Table 5). Since most of the antinematodal agents (e.g. Morantel tartrate, emamectin benzoate) are known to block the muscle activity [9], HWY-4213 appears unique as it is predicted to be an acute calcium channel blocker.

In summary, we report the development of a HTS method and identify HWY-4213 as a dual acting nematocidal and fungicidal compound that promises a commercially application. Further identification and verification of HWY-4213 functional targets as well as product optimization, analog development, and formulation development are warranted.

## Materials and Methods

### Growth and Maintenance for Nematodes and Fungi

*B. xylophilus* YB-1 isolate and *Botrytis cinerea* were obtained from the Southern Forest Research Center of the Korea Forest Research Institute. *B. cinerea* was cultured on 100-mm potato dextrose agar (PDA) plates at 25°C for 5 days as described. *B. xylophilus* YB-1 were reared by feeding *B. cinerea* grown on PDA plates at 25°C for 7 days and isolated using Baermann Funnel techniques [24]. *C. elegans* and *C. briggsae* were obtained from the *Caenorhabditis* Genetics Center (Twin Cities, Minnesota). Wild-type *C. elegans* and *C. briggsae* strains were maintained at strain-specific optimal temperatures on nematode growth media (NGM) agar plates seeded with *E. coli* OP50 as described by Brenner [25]. Blue stain fungus (*O. minus*) was obtained from Korea Agricultural Culture Collection (KACC), and was cultured on 100-mm malt extract agar (MEA) plates at 25°C. *Aspergillus flavus* and *A. fumigatus* were obtained from Dr. Jae-Kwan Hwang (Yonsei University, Seoul).

### Chemicals, Solubilizers, and Solvents

HWY-4213(1-*n*-undecyl-2-(2-fluorphenyl)methyl-3,4-dihydro-6,7-dimethoxy isoquinolinium chloride, C_29_H_41_ClFNO_2_, M.W. 490.11) and other HWY compounds were from Hanwha Chemical Research & Development Center (Taejon, Korea). HWY-4213 used throughout this study contains >98% purity and its chemical characteristics are as follows: ^1^HNMR (CDCl_3_, 300 MHz) 0.88 (t, *J* = 6.3 Hz, 3H), 1.18∼1.36 (m, 14H), 1.40∼1.50 (m, 2H), 1.56∼1.72 (m, 2H), 3.17 (t, *J* = 7.5 Hz, 2H), 3.32 (t, *J* = 8.1 Hz, 2H), 3.93 (s, 3H), 4.00 (s, 3H), 4.18 (t, *J* = 6.9 Hz, 2H), 5.77 (s, 2H), 6.80 (s, 1H), 7.10 (t, *J* = 9.0 Hz, 1H), 7.22 (s, 1H), 7.25 (m, 1H), 7.41 (m, 1H), 7.95 (t, *J* = 7.2 Hz, 1H). Other chemicals (HWY = series compounds) are mostly of >98% purity (provided by Hanwha Chemical Research and Development, Taejon, Korea). Abamectin, emamectin benzoate, levamisol, morantel tartrate, amphotericin B, itraconazole, ketoconazole, methyl ethyl ketone (MEK) and Ca^2+^ channel blockers were purchased from Merck (New Jersey, USA) and Sigma (St Louis, MO, USA). Fluconazole was obtained from Yuhan Pharmacent. Co. (Seoul, Korea). Polyoxyethylene (40, 50, and 60) condensates of hydrogenated castor oils (Nikko Chemical, Tokyo, Japan) were used as solubilizers for emamectin benzoate and other commercially available compounds used in this study [9].

### Measurement of Nematode's Life Cycle, Life Span, and Brood Size

*B. xylophilus* life cycle, life span and brood size were measured in worms grown on plates of *Botrytis cinerea* cultured in advance on a 50-mm observation plate that prevents overgrowth of fungal hypha and allows easy microscopic observation. To measure the life cycle, 100 adult female *B. xylophilus* were placed on an observation plate and allowed to lay eggs for 3 h at 25°C. Eggs were observed for hatching, molting and the morphology using the differential interference contrast (Nomarski) microscopy. For mating, one female and three males were placed on one observation plate and cultured at 25°C, and eggs and brood size were measured [26]. Large amount of eggs were collected using watch glasses [14].

### Establishment of the HTS Method and Compound Identification

The CBA method, a reference screening method, was performed to determine IC_50_. Compounds were first dissolved in dimethylsulfoxide (DMSO) to a concentration of 200 mM. The final concentration of DMSO is 1% (w/v). Nematodes were divided into 96 well plates and treated with 2 µl of the drug with the final concentrations of 2 mM, 1 mM, 0.1 mM, 0.01 mM, and 0.001 mM. Polyoxyethylene was used only when crystals were evident upon mixing of a stock solution with water (less than 10% w/w). The subcultured *B. xylophilus* was separated using the Baermann funnel technique and diluted to 20,000 nematodes/mL. Both the *B. xylophilus* solution (50% of the total volume) and drug stock solution of (1% of total volume) were aliquoted into 96-well microplates (e.g., 100 µl *B. xylophilus* and 2 µl 200 mM stock solution of test compounds in a total volume of 200((l). An aliquot of *B. xylophilus* and the drug were incubated at 25(C for 6 h, after which 20((l aliquot was transferred to a blank 50-mm agar plate and allowed to stand at room temperature for 10 min. The total number of live and dead worms was counted to assess LD50 or the relative nematicidal activity. All experiments, including *in vitro* assays, were performed three times in duplicate unless otherwise specified.

### Broth Microdilution Test of Antifungal Susceptibility

Inoculum suspensions of filamentous fungi and commercial antifungal agents were prepared by the method of National Committee for Clinical Laboratory Standards (NCCLS) M38-A (NCCLS, 2002) [27]. Antifungal susceptibility tests were used to determine the MIC and MFC of each compound. Individual MICs and MFCs were determined following the broth microdilution method as recommended by the NCCLS, following the approved standard M38-A (NCCLS, 2002) as modified [27]–[29].

### Preliminary Evaluation of Preventative and Therapeutic Effects of HWY-4213 against Pot-Grown Pine Trees

Pot-grown 4 year-old seedlings of *Pinus densiflora* (average height: 40 cm; average basal diameter: 1.5 cm) were purchased from Sang Ju Farm (Sangju, Korea). The preventative effect of HWY-4213 was tested by injecting HWY-4213 into the wood 2 weeks prior to inoculating with *B. xylophilus* as previously described [10]. The therapeutic effect of HWY-4213 was tested by first inoculating trees with *B. xylophilus*, then injecting HWY-4213 two weeks later. The dead seedlings were cross-cut and examined for the presence of dehydrated areas as evidence of disease [10]. The number of branches at the uppermost joint of all inoculated specimens was counted. PWNs in tissue segments collected from the middle portion of the main stems and the base of the stems were extracted by the Baermann funnel technique [24] and counted under a dissecting microscope. The feeding dose was expressed as the weight (g) of HWY-4213 per unit volume (m3) of the tree. Assuming that the average volume corresponds to twice the volume of a cone, the volume of a test tree was calculated as described [10].

## Supporting Information

Table S1 Anti-nematodal activity of HWY-4213 against various nematodes. *Values are mean±SD, from three independent experiments (n = 3).
(0.03 MB DOC)

Table S2 Selection of solvent for trunk-injection agent. Solubility tests employed three solvents: MEK, methanol and acetone. Water solubility of HWY 4213/solvent solutions were determined by mixing HWY 4213 (100 mg) and solvent (100–300 µL) in distilled water (1 mL) and then 1) standing at −20°C for 7 days, or 2) standing and at room temperature (20°C) for 72 h. To test solubility in resin, HWY-4213 (100 mg) and solvent (100 300 µL) were added to resin (10–100 mg) and shaken at room temperature for 24 h. Solubility of each formulation using each preparation method was determined by visually inspecting for formation of a precipitate.
(0.04 MB DOC)

Table S3 Test of injection speed. This data represents the time required to inject 20 mL of the trunk-injection form of HWY-4213 into the wood in August, a time of high pine wood resin excretion. Resin excretion prevents injection of Morantel tartrate and Emamectin Benzoate into the wood of *P. densiflora*.
(0.03 MB DOC)

Table S4 Relative sensitivity of HWY-4213 to *C. elegans* mutants deficient in channel proteins. *Values are mean±SD, from three independent experiments (n = 3).
(0.09 MB DOC)

Text S1 Selection of solvent for trunk-injection agent.
(0.03 MB DOC)

Figure S1 The eggs were observed for time of hatching, molting and morphology using differential interference contrast (Nomarski) microscopy. (A) 4 cell stage, (B) J1 stage in egg, (C) J2 stage in egg, (D) J2 stage after hatching, (E) Propagative J3 stage, (F) Oil red O staining of propagative J3 stage (G) Dispersal J3 stage, (H) Oil red O staining of dispersal J3 stage, (I) Male J4 stage (red box is gonad position), (J) Female J4 stage (red box is gonad position), (K) Gonad of male J4 stage (L) Gonad of female J4 stage, (M) Male adult stage (red box is gonad in the head portion) (N) Female adult stage (red box is gonad in the head portion), (O) Gonad of male adult stag (P) Gonad of female adult stage. Scale bars; A–D and O–P = 20 µm, E–H = 50 µm, I–J and M–N = 100 µm, K–L = 10 µm.
(1.28 MB TIF)

Figure S2 Life span of YB-1 isolates of *B. xylophilus*. Life span was determined for both mated and unmated *B. xylophilus*. (A) In the continuously mated group, the maximum life span at 25°C was 30–35 days; the mean life span was 17 days for males and 18 days for females. (B) In the unmated group, the maximum life span for both sexes was 70 days. The mean life span of unmated males and females was 45 days and 51 days, respectively, or approximately twice as long as that in the mated group.
(0.14 MB TIF)

Figure S3 The *in vivo* test of HWY-4213 efficacy The *in vivo* efficacy of HWY-4213 was tested using the pot-grown 4-year-old seedlings of *Pinus densiflora* (average height: 40 cm; average basal diameter: 1.5 cm) as described in “Materials and Methods”. Shown here are the HWY-4213 treated seedlings at 15-day post injection (A) and control seedlings that received only vehicle solution (B).
(2.14 MB TIF)

Figure S4 Comparison of morphological change of dead worms treated with Ca^2+^ channel blocker assay. The morphological view of the dead worms (pharynx) who received 0.1 mM HWY-4213 (A), Miberfradil (T-type Ca^2+^ channel blocker, 1 mM) (B) and Verpamil (L-type Ca^2+^ channel blocker, 1 mM) (C). (scale bar: 10 µm, x400).
(1.79 MB TIF)
