# Supplementary material for: Identification and Characterization of a Dual-Acting Antinematodal Agent against the Pinewood Nematode, Bursaphelenchus xylophilus
Source: PLoS One. 2009 Nov 11;4(11):e7593. doi: 10.1371/journal.pone.0007593 (PMC2771284; doi:10.1371/journal.pone.0007593)
Supplement: Table S1 — Anti-nematodal activity of HWY-4213 against various nematodes. *Values are mean±SD, from three independent experiments (n = 3). (0.03 MB DOC) [file pone.0007593.s001.doc]

|  | **HWY-4213*** **(mM)** |  |  |
| --- | --- | --- | --- |
|  | **LD25** | **LD50** | **LD95** |
| ***B. xylophilus*** | 0.171 (± 0.089) | 0.447 (± 0.079) | 0.944 (± 0.002) |
| ***C. elegans*** | 0.082 (± 0.059) | 0.143 (± 0.020) | 0.591 (± 0.002) |
| ***C. briggsae*** | 0.076 (± 0.042) | 0.151 (± 0.025) | 0.503 (± 0.002) |
