# Supplementary material for: Identification and Characterization of a Dual-Acting Antinematodal Agent against the Pinewood Nematode, Bursaphelenchus xylophilus
Source: PLoS One. 2009 Nov 11;4(11):e7593. doi: 10.1371/journal.pone.0007593 (PMC2771284; doi:10.1371/journal.pone.0007593)
Supplement: Table S2 — Selection of solvent for trunk-injection agent. Solubility tests employed three solvents: MEK, methanol and acetone. Water solubility of HWY 4213/solvent solutions were determined by mixing HWY 4213 (100 mg) and solvent (100–300 µL) in distilled water (1 mL) and then 1) standing at −20°C for 7 days, or 2) standing and at room temperature (20°C) for 72 h. To test solubility in resin, HWY-4213 (100 mg) and solvent (100 300 µL) were added to resin (10–100 mg) and shaken at room temperature for 24 h. Solubility of each formulation using each preparation method was determined by visually inspecting for formation of a precipitate. (0.04 MB DOC) [file pone.0007593.s002.doc]

| **Compounds (μL)** | **1** | **2** | **3** | **4** | **5** | **6** | **7** | **8** | **9** | **10** |
| --- | --- | --- | --- | --- | --- | --- | --- | --- | --- | --- |
| **HWY-4213** | 100 | 100 | 100 | 100 | 100 | 100 | 100 | 100 | 100 | 100 |
| **Methanol** | 100 | 200 | 300 | - | - | - | - | - | - | - |
| **MEK** | - | - | - | 100 | 200 | 300 | - | - | - | - |
| **Acetone** | - | - | - | - | - | - | 100 | 200 | 300 | - |
| **D.W.** | 800 | 700 | 600 | 800 | 700 | 600 | 800 | 700 | 600 | 900 |
| **Total** | 1000 | 1000 | 1000 | 1000 | 1000 | 1000 | 1000 | 1000 | 1000 | 1000 |
| **Appearance (20ºC, 0 h)** | No | No | No | No | No | No | No | No | No | No |
| **Appearance (20ºC, 72 h)** | No | No | No | No | No | No | No | No | No | No |
| **Freeze (-20ºC, 7 d)** | Yes | No | No | No | No | No | Yes | Yes | Yes | Yes |
| **Resin solubility (mg)** | 0 | 0 | 0 | 10 | 60 | 100 | 0 | 10 | 10 | 0 |
