# Supplementary material for: Identification and Characterization of a Dual-Acting Antinematodal Agent against the Pinewood Nematode, Bursaphelenchus xylophilus
Source: PLoS One. 2009 Nov 11;4(11):e7593. doi: 10.1371/journal.pone.0007593 (PMC2771284; doi:10.1371/journal.pone.0007593)
Supplement: Table S3 — Test of injection speed. This data represents the time required to inject 20 mL of the trunk-injection form of HWY-4213 into the wood in August, a time of high pine wood resin excretion. Resin excretion prevents injection of Morantel tartrate and Emamectin Benzoate into the wood of P. densiflora. (0.03 MB DOC) [file pone.0007593.s003.doc]

| **Compounds (in 20 mL vol.)** | **Injection time (second)** |  |  | **Average (second) (mean)** |
| --- | --- | --- | --- | --- |
|  | **1^st^** | **2^nd^** | **3^rd^** |  |
| **HWY-4213 (in 10% MEK)** | 578 | 597 | 554 | 576.3 |
| **HWY-4213 (in 20% MEK)** | 321 | 312 | 281 | 304.7 |
| **HWY-4213 (in 30% MEK)** | 300 | 291 | 311 | 300.6 |
| **HWY-4213** | 642 | 612 | 698 | 617.3 |
| **Morantel tartrate** | N.D. | N.D. | N.D. | N.D. |
| **Emamectin Benzoate** | N.D. | N.D. | N.D. | N.D. |
