# Supplementary material for: Identification and Characterization of a Dual-Acting Antinematodal Agent against the Pinewood Nematode, Bursaphelenchus xylophilus
Source: PLoS One. 2009 Nov 11;4(11):e7593. doi: 10.1371/journal.pone.0007593 (PMC2771284; doi:10.1371/journal.pone.0007593)
Supplement: Table S4 — Relative sensitivity of HWY-4213 to C. elegans mutants deficient in channel proteins. *Values are mean±SD, from three independent experiments (n = 3). (0.09 MB DOC) [file pone.0007593.s004.doc]

| **No.** | **CGC No.** | **Stain** | **Genotype** | **Lethality by HWY-4213 (%)*** |  |  |
| --- | --- | --- | --- | --- | --- | --- |
|  |  |  |  | **0.01 mM** | **0.1 mM** | **1 mM** |
| **1** |  | N2 |  | 5.3 (±1.8) | 92.8 (±4.3) | 100 (±0.0) |
| **2** | RB918 | *acr-16* | *acr-16(ok789) V* | 6.4 (±3.4) | 75.8 (±10.7) | 100 (±0.0) |
| **3** | RB1659 | *acr-3* | *acr-3(ok2049) X* | 5.1 (±3.9) | 93.4 (±4.7) | 100 (±0.0) |
| **4** | DA1051 | *avr-15* | *avr-15(ad1051) V* | 4.9 (±1.5) | 96.4 (±2.7) | 100 (±0.0) |
| **5** | JD21 | *cca-1* | *cca-1(ad1650) X* | 5.8 (±3.4) | 19.6 (±5.2) | 100 (±0.0) |
| **6** | RB1310. | *clc-2* | *clc-2(ok1426) X* | 5.8 (±3.7) | 90.5 (±5.7) | 100 (±0.0) |
| **7** | XA900 | *clh-1* | *clh-1(qa900) II* | 6.3 (±3.1) | 74.5 (±7.6) | 100 (±0.0) |
| **8** | RB920 | *clh-6* | *clh-6(ok791) V* | 5.9 (±4.3) | 59.3 (±8.7) | 100 (±0.0) |
| **9** | VC220 | *cmk-1* | *cmk-1(ok287) IV* | 5.0 (±3.1) | 63.4 (±14.2) | 100 (±0.0) |
| **10** | KJ462 | *cng-3* | *cng-3(jh113) IV* | 5.6(±3.0) | 38.4 (±6.7) | 100 (±0.0) |
| **11** | TU1366 | *deg-1* | *deg-1(u506)X* | 3.2(±2.5) | 79.6 (±5.9) | 100 (±0.0) |
| **12** | TU1747 | *deg-3* | *deg-3(u662)V* | 3.8 (±2.0) | 91.3 (±3.3) | 100 (±0.0) |
| **13** | NC279 | *del-1* | *del-1(ok150) X* | 3.0 (±2.1) | 77.8 (±10.1) | 100 (±0.0) |
| **14** | DA453 | *eat-2* | *eat-2(ad453)II* | 3.0 (±2.2) | 71.1 (±5.7) | 98.7 (±2.2) |
| **15** | DA1013 | *egl-19* | *egl-19(ad1013) IV* | 3.8 (±2.0) | 93.4 (±3.0) | 100 (±0.0) |
| **16** | MT1444 | *egl-2* | *egl-2(n693) V* | 2.2 (±1.7) | 71.5 (±5.4) | 100 (±0.0) |
| **17** | NJ469 | *exc-4* | *exc-4(rh133)I* | 4.9 (±4.1) | 81.9 (±4.8) | 100 (±0.0) |
| **18** | DA1426 | *exp-2* | *exp-2(sa26ad1426) V* | 6.5 (±1.8) | 88.0 (±3.5) | 100 (±0.0) |
| **19** | JD31 | *glc-4* | *glc-4(ok212) II* | 5.4 (±1.3) | 79.3 (±5.3) | 100 (±0.0) |
| **20** | VC350 | *glc-2* | *glc-2(gk179) I* | 2.7 (±2.3) | 93.5 (±4.7) | 100 (±0.0) |
| **21** | KP4 | *glr-1* | *glr-1(n2461)III* | 9.9 (±4.1) | 95.5 (±2.9) | 100 (±0.0) |
| **22** | VC244 | *gtl-1* | *gtl-1(ok375) IV* | 4.2 (±3.9) | 95.4 (±2.0) | 100 (±0.0) |
| **23** | RB1808 | *gtl-2* | *gtl-2(ok2342) III* | 5.4 (±4.5) | 89.6 (±4.9) | 100 (±0.0) |
| **24** | VC994 | *inx-9* | *inx-9(ok1502) IV* | 4.6 (±3.8) | 81.2 (±7.4) | 100 (±0.0) |
| **25** | RB883 | *kqt-2* | *kqt-2(ok732) X* | 5.5 (±2.4) | 74.3 (±4.3) | 100 (±0.0) |
| **26** | CB1292 | *mec-1* | *mec-1(e1292)V* | 6.2 (±3.3) | 91.1 (±3.8) | 100 (±0.0) |
| **27** | TU55 | *mec-14* | *mec-14(u55)III* | 4.4 (±3.5) | 85.1 (±6.7) | 100 (±0.0) |
| **28** | CB1339 | *mec-4* | *mec-4(e1339)X* | 5.3 (±2.1) | 91.8 (±4.4) | 100 (±0.0) |
| **29** | VC9 | *nca-2* | *nca-2(gk5) III* | 1.4 (±1.3) | 17.8 (±6.7) | 99.2 (±1.2) |
| **30** | VC1233 | *ocr-2* | *ocr-2(ok1711) IV* | 3.6 (±2.8) | 90.1 (±3.5) | 100 (±0.0) |
| **31** | LY100 | *slo-2* | *slo-2(nf100) X* | 4.6 (±3.1) | 81.9 (±2.9) | 99.4 (±1.0) |
| **32** | MT180 | *sup-9* | *sup-9(n180)II* | 4.3 (±3.2) | 91.7 (±6.9) | 100 (±0.0) |
| **33** | RB1095 | *tag-130* | *tag-130(ok1073)X* | 5.7 (±4.2) | 93.2 (±3.5) | 100 (±0.0) |
| **34** | PR671 | *tax-2* | *tax-2(p671)I* | 1.1 (±1.1) | 86.6 (±8.1) | 99.7 (±0.6) |
| **35** | FK104 | *tax-2* | *tax-2(ks31)I* | 3.6 (±3.0) | 77.2 (±4.2) | 100 (±0.0) |
| **36** | FK129 | *tax-4* | *tax-4(ks11)I* | 8.1 (±3.5) | 96.6 (±2.9) | 100 (±0.0) |
| **37** | CB1597 | *unc-103* | *unc-103(e1597)III* | 2.8 (±2.3) | 73.3 (±6.3) | 100 (±0.0) |
| **38** | RB1316 | *unc-105* | *unc-105(ok1432) II* | 5.0 (±3.5) | 20.8 (±6.4) | 100 (±0.0) |
| **39** | CB15 | *unc-8* | *unc-8(e15)IV* | 4.3 (±3.1) | 82.1 (±9.3) | 100 (±0.0) |
| **40** | CB101 | *unc-9* | *unc-9(e101)X* | 3.4 (±2.7) | 18.7 (±5.3) | 100 (±0.0) |
