# Supplementary material for: Identification and Characterization of a Dual-Acting Antinematodal Agent against the Pinewood Nematode, Bursaphelenchus xylophilus
Source: PLoS One. 2009 Nov 11;4(11):e7593. doi: 10.1371/journal.pone.0007593 (PMC2771284; doi:10.1371/journal.pone.0007593)
Supplement: Text S1 — Selection of solvent for trunk-injection agent. (0.03 MB DOC) [file pone.0007593.s005.doc]

**Supplementary Text S1**

**Selection of Solvent for Trunk-Injection Agent.**

To select a potential carrier solution for HWY-4213, we tested three solvents: methyl ethyl ketone (MEK), methanol and acetone. Solubility tests employed three solvents: MEK, methanol and acetone. Water solubility of HWY‑4213/solvent solutions were determined by mixing HWY‑4213 (100 mg) and solvent (100–300 μL) in distilled water (1 mL) and then 1) standing at -20ºC for 7 days, or 2) standing at room temperature (20ºC) for 72 h. To test solubility in resin, HWY-4213 (100 mg) and solvent (100‑300 μL) were added to resin (10-100 mg) and shaken at room temperature for 24 h. Solubility of each formulation using each preparation method was determined by visually inspecting for formation of a precipitate. Infiltration rate into wood was measured in pine trees (*Pinus densiflora*) by injecting 20 mL of test drug solution with a needle and syringe.
