# Supplementary figures and images for: Identification and Characterization of a Dual-Acting Antinematodal Agent against the Pinewood Nematode, Bursaphelenchus xylophilus
Source: PLoS One. 2009 Nov 11;4(11):e7593. doi: 10.1371/journal.pone.0007593 (PMC2771284; doi:10.1371/journal.pone.0007593)

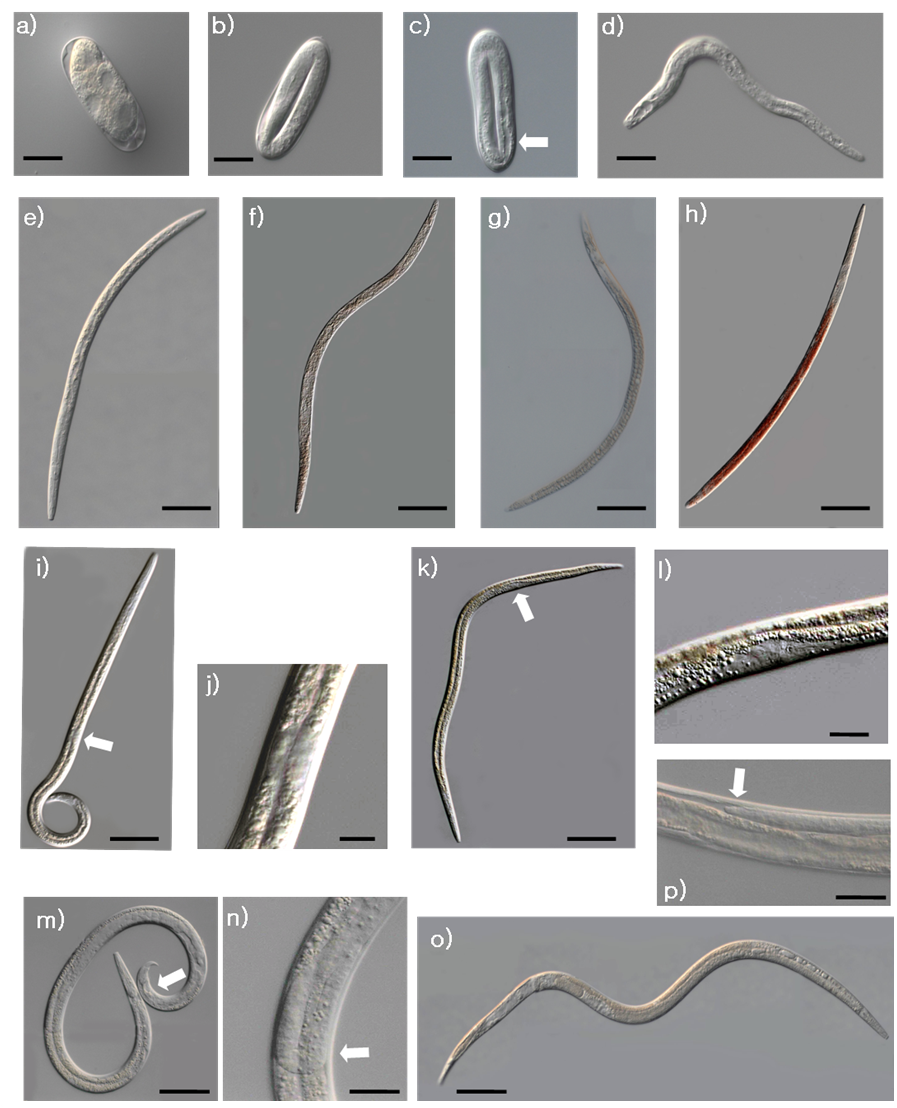

Supplement: Figure S1 — The eggs were observed for time of hatching, molting and morphology using differential interference contrast (Nomarski) microscopy. (A) 4 cell stage, (B) J1 stage in egg, (C) J2 stage in egg, (D) J2 stage after hatching, (E) Propagative J3 stage, (F) Oil red O staining of propagative J3 stage (G) Dispersal J3 stage, (H) Oil red O staining of dispersal J3 stage, (I) Male J4 stage (red box is gonad position), (J) Female J4 stage (red box is gonad position), (K) Gonad of male J4 stage (L) Gonad of female J4 stage, (M) Male adult stage (red box is gonad in the head portion) (N) Female adult stage (red box is gonad in the head portion), (O) Gonad of male adult stag (P) Gonad of female adult stage. Scale bars; A–D and O–P = 20 µm, E–H = 50 µm, I–J and M–N = 100 µm, K–L = 10 µm. (1.28 MB TIF) [file pone.0007593.s006.tif]

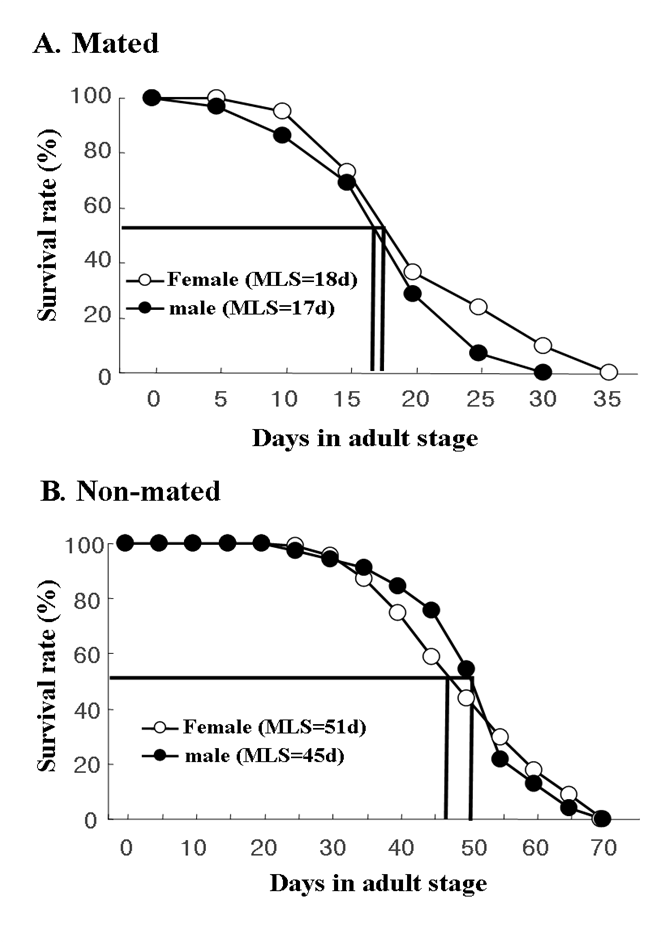

Supplement: Figure S2 — Life span of YB-1 isolates of B. xylophilus. Life span was determined for both mated and unmated B. xylophilus. (A) In the continuously mated group, the maximum life span at 25°C was 30–35 days; the mean life span was 17 days for males and 18 days for females. (B) In the unmated group, the maximum life span for both sexes was 70 days. The mean life span of unmated males and females was 45 days and 51 days, respectively, or approximately twice as long as that in the mated group. (0.14 MB TIF) [file pone.0007593.s007.tif]

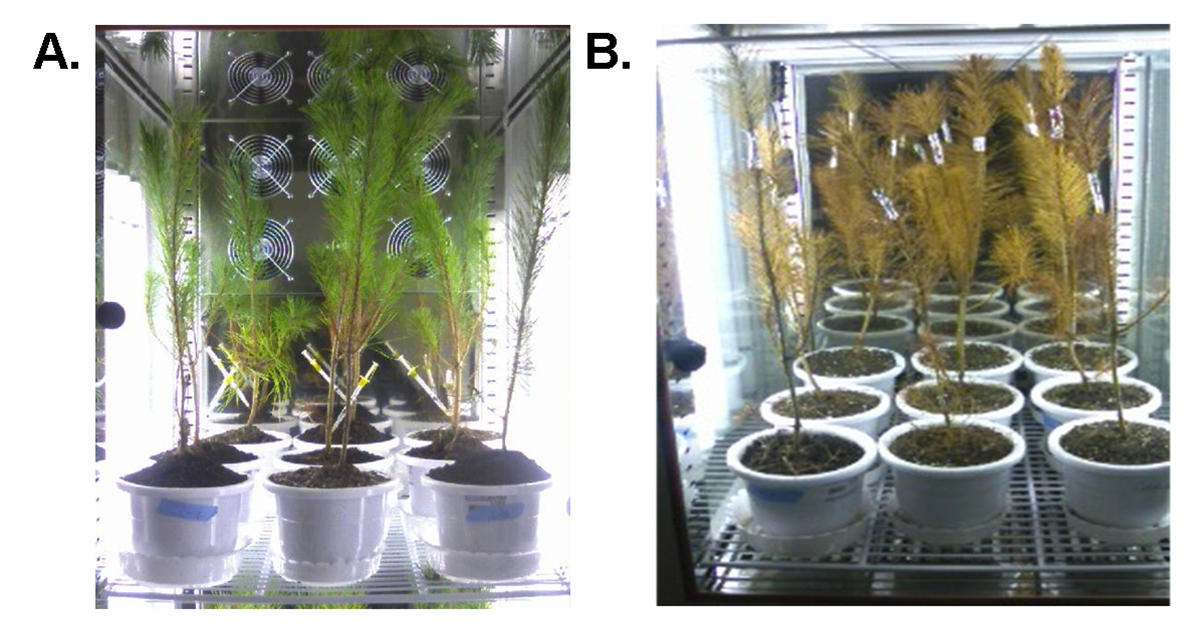

Supplement: Figure S3 — The in vivo test of HWY-4213 efficacy The in vivo efficacy of HWY-4213 was tested using the pot-grown 4-year-old seedlings of Pinus densiflora (average height: 40 cm; average basal diameter: 1.5 cm) as described in “Materials and Methods”. Shown here are the HWY-4213 treated seedlings at 15-day post injection (A) and control seedlings that received only vehicle solution (B). (2.14 MB TIF) [file pone.0007593.s008.tif]

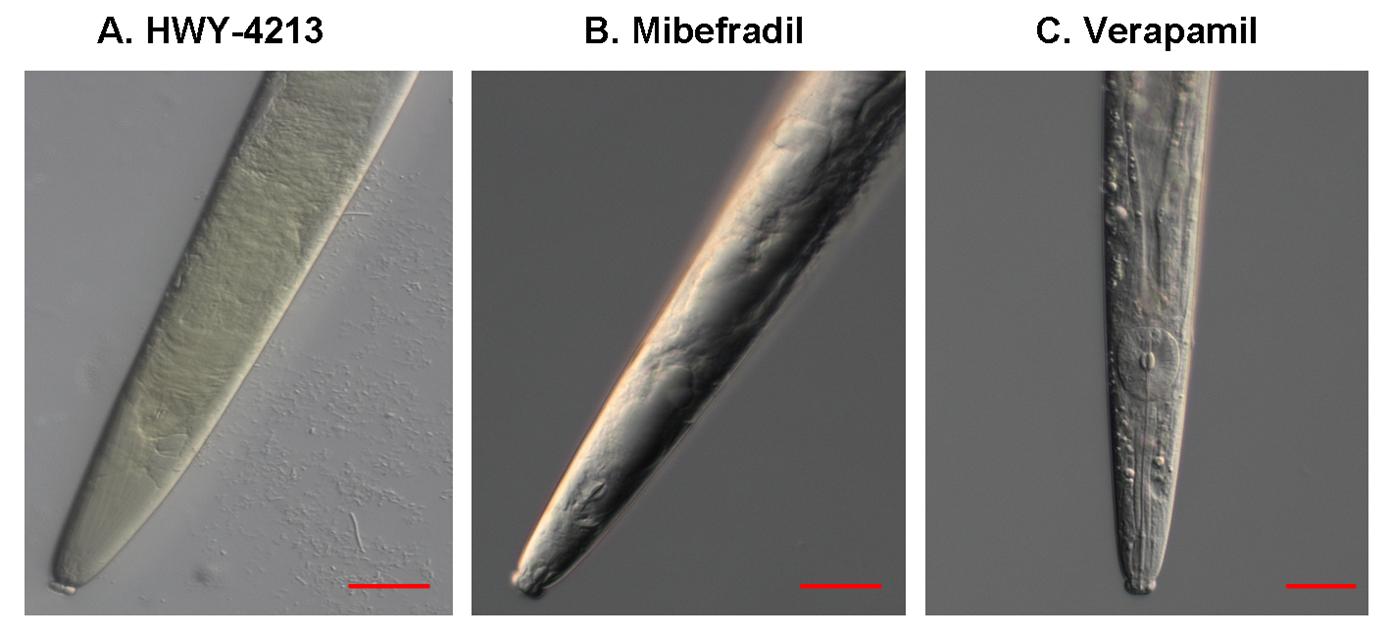

Supplement: Figure S4 — Comparison of morphological change of dead worms treated with Ca2+ channel blocker assay. The morphological view of the dead worms (pharynx) who received 0.1 mM HWY-4213 (A), Miberfradil (T-type Ca2+ channel blocker, 1 mM) (B) and Verpamil (L-type Ca2+ channel blocker, 1 mM) (C). (scale bar: 10 µm, x400). (1.79 MB TIF) [file pone.0007593.s009.tif]
